# Molecular Pathogenesis and Therapy of Polycythemia Induced in Mice by JAK2 V617F

**DOI:** 10.1371/journal.pone.0000018

**Published:** 2006-12-20

**Authors:** Virginia M. Zaleskas, Daniela S. Krause, Katherine Lazarides, Nihal Patel, Yiguo Hu, Shaoguang Li, Richard A. Van Etten

**Affiliations:** 1 Molecular Oncology Research Institute, Tufts‐New England Medical Center Boston, Massachusetts, United States of America; 2 The Jackson Laboratory Bar Harbor, Maine, United States of America; Memorial Sloan-Kettering Cancer Center, United States of America

## Abstract

**Background:**

A somatic activating mutation (V617F) in the JAK2 tyrosine kinase was recently discovered in the majority of patients with polycythemia vera (PV), and some with essential thrombocythemia (ET) and chronic idiopathic myelofibrosis. However, the role of mutant JAK2 in disease pathogenesis is unclear.

**Methods and Findings:**

We expressed murine JAK2 WT or V617F via retroviral bone marrow transduction/transplantation in the hematopoietic system of two different inbred mouse strains, Balb/c and C57Bl/6 (B6). In both strains, JAK2 V617F, but not JAK2 WT, induced non-fatal polycythemia characterized by increased hematocrit and hemoglobin, reticulocytosis, splenomegaly, low plasma erythropoietin (Epo), and Epo-independent erythroid colonies. JAK2 V617F also induced leukocytosis and neutrophilia that was much more prominent in Balb/c mice than in B6. Platelet counts were not affected in either strain despite expression of JAK2 V617F in megakaryocytes and markedly prolonged tail bleeding times. The polycythemia tended to resolve after several months, coincident with increased spleen and marrow fibrosis, but was resurrected by transplantation to secondary recipients. Using donor mice with mutations in Lyn, Hck, and Fgr, we demonstrated that the polycythemia was independent of Src kinases. Polycythemia and reticulocytosis responded to treatment with imatinib or a JAK2 inhibitor, but were unresponsive to the Src inhibitor dasatinib.

**Conclusions:**

These findings demonstrate that JAK2 V617F induces Epo-independent expansion of the erythroid lineage in vivo. The fact that the central erythroid features of PV are recapitulated by expression of JAK2 V617F argues that it is the primary and direct cause of human PV. The lack of thrombocytosis suggests that additional events may be required for JAK2 V617F to cause ET, but qualitative platelet abnormalities induced by JAK2 V617F may contribute to the hemostatic complications of PV. Despite the role of Src kinases in Epo signaling, our studies predict that Src inhibitors will be ineffective for therapy of PV. However, we provide proof-of-principle that a JAK2 inhibitor should have therapeutic effects on the polycythemia, and perhaps myelofibrosis and hemostatic abnormalities, suffered by MPD patients carrying the JAK2 V617F mutation.

## Introduction

The myeloproliferative diseases (MPDs) chronic myeloid leukemia (CML), polycythemia vera (PV), essential thrombocythemia (ET), and chronic idiopathic myelofibrosis (CIMF) are clonal disorders characterized by overproduction of mature myeloerythroid cells, abnormalities of hemostasis and thrombosis, and tendency to progress to acute leukemia [1], [2]. The cause of CML is the product of the Philadelphia chromosome, the BCR-ABL fusion tyrosine kinase. Retroviral expression of BCR-ABL in murine bone marrow (BM) causes CML-like MPD with overproduction of maturing neutrophils [3], whereas the BCR-ABL kinase inhibitor imatinib induces hematologic and cytogenetic remissions in CML patients [4].

By contrast, the pathogenesis of the other MPDs is less clear. PV is characterized by overproduction of mature erythrocytes, increased hematocrit and red cell mass, and splenomegaly due to extramedullary hematopoiesis [5]. Many PV patients also have increased circulating granulocytes and platelets. PV can be complicated by abnormalities of hemostasis, including platelet dysfunction and bleeding, as well as arterial and venous thrombosis. The disease infrequently evolves to acute myeloid leukemia, while progression to a “spent” phase, characterized by myelofibrosis and normal or low hematocrit, is more common. A hallmark of PV is the presence of endogenous erythroid colonies (EEC), erythroid progenitors that form colonies in vitro in the absence of exogenous erythropoietin (Epo) [6], but demonstrate hypersensitivity to insulin-like growth factor-1 [7]. Biochemical and molecular studies of PV patients have revealed no mutations in the Epo receptor, but granulocytes from PV patients have increased transcripts for the urokinase plasminogen activator receptor family member PRV-1 [8], whereas PV platelets show decreased expression of c-Mpl, the receptor for thrombopoietin [9]. Abnormally increased tyrosine phosphatase activity has also been characterized in erythroid progenitors in PV [10]. However, whether these abnormalities are fundamental to the pathogenesis of PV was unclear.

JAK2 is a member of the Janus family of non-receptor tyrosine kinases, and is required for signaling from the Epo receptor and other type I cytokine receptors [11]. Recently, a somatic mutation in the JAK2 tyrosine kinase was identified in MPD patients. Studies of erythroid progenitors from PV patients demonstrated that Epo-independent erythroid maturation was impaired by a JAK2 inhibitor [12] and by siRNA knockdown of JAK2 [13]. This prompted sequencing of the JAK2 gene, which identified a G to A point mutation, resulting in substitution of phenylalanine for valine at amino acid 617 (V617F) in the JAK2 pseudokinase domain in the majority of PV patients [13]. The JAK2 V617F mutant had constitutive kinase activity in vivo in the absence of Epo stimulation, and retroviral expression in murine BM caused erythrocytosis [13]. The same mutation was independently identified through genomic sequencing of tyrosine kinases in MPD patients [14], [15], and by investigation of loss of heterozygosity involving the JAK2 gene on chromosome 9p [16]. The JAK2 V617F mutation is found in nearly every patient with PV and is present in homozygous form through mitotic recombination in up to 30% of patients. The mutation is also found in 30–60% of ET and CIMF patients, but is rarely found outside MPD [17]–[19].

The widespread finding of JAK2 V617F in the non-CML MPDs suggests that it may contribute to the pathogenesis of these diseases. However, it is not clear whether JAK2 V617F can be implicated as the direct and primary cause of PV, ET, or CIMF, nor is the relationship between the different MPDs that share the JAK2 mutation understood. Here, we investigated the role of JAK2 V617F in the pathogenesis of MPD by expression of the mutant kinase in the hematopoietic system of laboratory mice, using retroviral BM transduction and transplantation. Our results suggest that JAK2 V617F is the primary cause of PV, but not of ET. We also used this model system to study the signaling pathways in PV, and assess the response of the disease to kinase inhibitor therapy.

## Methods

### DNA constructs

The V617F mutation was generated in the murine JAK2 cDNA using PCR mutagenesis, verified by DNA sequencing, and the JAK2 WT or V617F mutant cDNAs cloned into the MIG R1 vector [20] in the position 5′ to the internal ribosome entry site.

### Retroviral BM transduction and transplantation

Generation and titering of replication-defective ecotropic retroviral stocks was done with the *kat* packaging system as previously described [21]. Retroviral titers were matched by flow cytometric analysis of GFP expression in transduced fibroblasts and primary BM cells. We performed retroviral BM transduction and transplantation as previously described [21], using donors pretreated with 5-fluorouracil (200 mg/kg) four days before harvest, and transplanting lethally irradiated recipients (900 cGy for Balb/c, 1050 cGy for B6). Balb/c and B6 mice were purchased from Taconic Farms; *Lyn* ^−/−^ *Hck* ^−/−^ *Fgr* ^−/−^ mice (>15 generations backcrossed into a B6 background) were the kind gift of Dr. Clifford Lowell [22]. Diseased mice were analyzed by histopathology and flow cytometric analysis as previously described [21]. Blood counts were determined by analysis of retro-orbital blood samples, anticoagulated with potassium EDTA, on a Hemavet 850 rodent hematology analyzer (Drew Scientific).

### Immunoblot analysis

Lysates were prepared from primary myeloerythroid cell suspensions from BM and spleen by direct boiling, fractionated by SDS-PAGE, and immunoblotted as previously described [21]. Antibodies against JAK2, Bcl-X_L_, and Src were obtained from Upstate Biotechnology, while antibodies against Gab2 were the kind gift of Haihua Gu (Beth Israel-Deaconess Hospital, Boston). All other antibodies, including anti-phospho-Tyr1007/1008 JAK2, anti-phospho-Tyr416 Src, anti-phospho and anti-pan Stat5, anti-phospho-Gab2, and phospho- and anti-pan ERK were obtained from Cell Signaling Technology.

### Southern blot analysis

The number of proviral clones in BM and spleen cells from mice with JAK2 V617F-induced MPD was determined by digesting genomic DNA with *BglII*, fractionation on agarose gels, transfer to nylon membranes, and hybridization with a radiolabeled probe from the *GFP* gene, as described [21].

### Analysis of erythrocyte lifespan and mass

Erythrocyte lifespan was determined by in vivo biotinylation of erythrocytes and serial flow cytometric detection of biotinylated red cells using streptavidin-PE, as described [23]. For red cell mass determination, approximately 0.5 ml of peripheral blood was obtained from a healthy donor mouse and biotinylated in vitro as described [23]. The concentration of erythrocytes was determined by counting on a Hemavet 850 hematology analyzer and the percentage of biotinylated erythrocytes determined by FACS. A defined volume (200–300 µl) was then injected into normal or polycythemic recipient mice, whose peripheral blood was sampled two hours later. The total RBC mass was calculated by the following formula: total RBC mass = (%labeling of donor RBC/100)×(#RBC/µL in donor sample)×(#µL injected)×100/(%labeled RBC in recipient).

### CFU-E and Epo assays

BM or spleen cells were plated in methylcellulose (MethoCult M3234, Stem Cell Technologies) with 5 ng/ml recombinant murine IL-3 (Peprotech) as a source of burst-promoting activity and varying concentrations of recombinant human Epo (Ortho). CFU-E colonies were identified on day 3 by morphology and diaminofluorine staining. Plasma Epo levels were determined by ELISA (R&D Systems).

### Tail bleeding time

Tail bleeding times were determined as previously described [24]. Briefly, following induction of isoflurane anesthesia, a 5 mm portion of the tail tip was severed by scalpel and the tip immersed in 0.9% saline solution at 37°C. The time of cessation of spontaneous bleeding was recorded; if bleeding continued for more than 15 minutes, the time was recorded as 900 seconds, and hemostasis achieved by cauterization of the tail tip.

### Purification of megakaryocytes

Megakaryocytes were purified after in vitro growth as previously described [25]. Briefly, BM or spleen cells were grown for 72–96 hours in IMDM with 1% Nutridoma (Roche) and 38 ng/ml recombinant human thrombopoietin (Peprotech), and megakaryocytes purified by sedimentation over a discontinuous albumin gradient. The resulting population was >95% megakaryocytes based on morphology and staining for CD41 and acetylcholinesterase.

### Drug treatments

Imatinib (obtained from the pharmacy) and dasatinib (kind gift of Drs. Frank Lee and Roberto Weinmann, Bristol-Myers Squibb) were dissolved in a water:propylene glycol (50:50) vehicle and administered by twice-daily oral gavage at 100 mg/kg [26] and 10 mg/kg [27] per dose, respectively. Control mice received vehicle alone. AG-490 (Calbiochem) was dissolved in 50% DMSO and delivered by subcutaneous osmotic minipump (Alzet model 2002) at a rate of about 300 µg per 24 hours, while control mice received minipumps loaded with 50% DMSO alone.

## Results

### JAK2 V617F induces polycythemia through Epo-independent overproduction of erythrocytes

**Figure 1:**
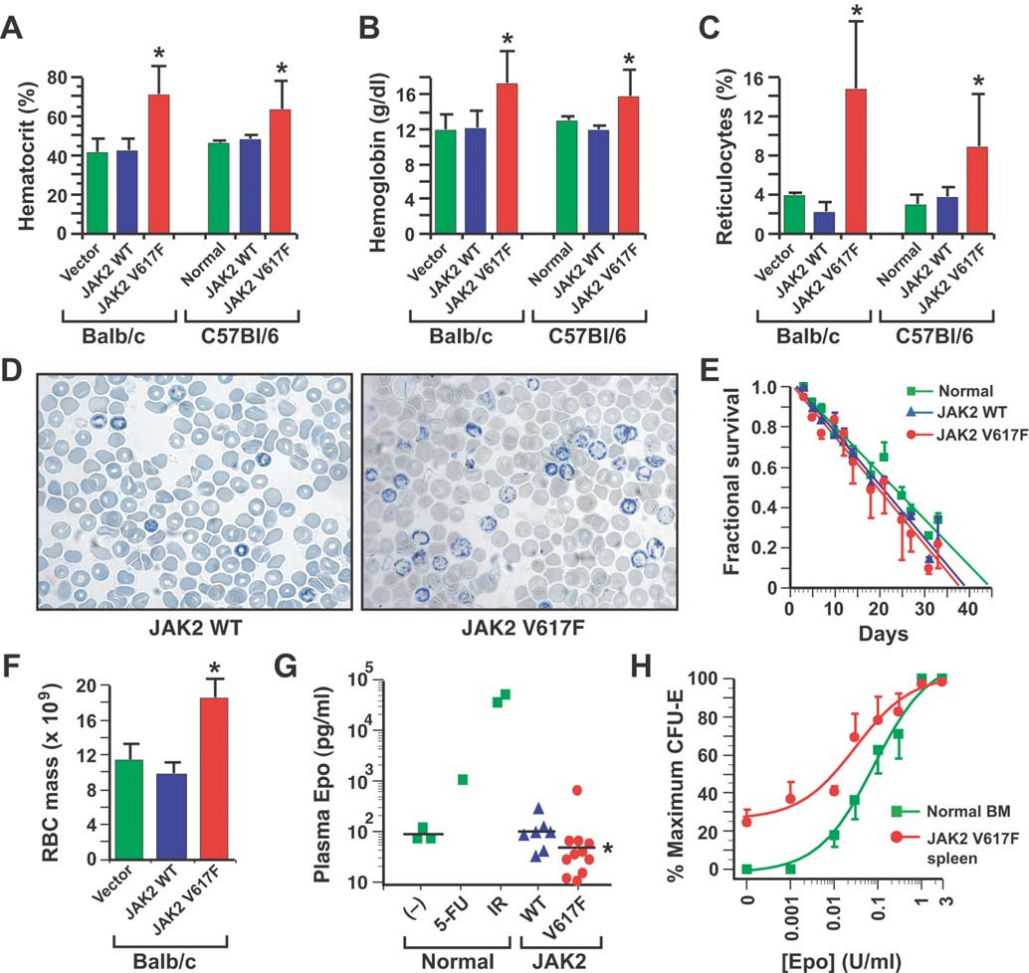
JAK2 V617F induces polycythemia through autonomous overproduction of erythrocytes. (A–C): Hematocrit (A), blood hemoglobin (B), and reticulocyte counts (C) from cohorts of Balb/c or B6 mice transplanted with syngeneic BM cells transduced with empty vector (green, *n* = 2), or retrovirus expressing murine JAK2 WT (blue, *n* = 10) or JAK2 V617F (red, *n* = 15). In the case of the B6 cohorts, untransplanted mice (“Normal”, *n* = 4) were used instead of empty vector controls. The difference between JAK2 V617F and JAK2 WT recipients was significant (unpaired *t*-test) for hematocrit (*P*<0.0001 for Balb/c and *P* = 0.0071 for B6), hemoglobin (*P* = 0.0011 for Balb/c and *P* = 0.0024 for B6), and reticulocytes (*P*<0.0001 for Balb/c and *P* = 0.0195 for B6), while there were no significant differences between vector and JAK2 WT recipients. (D) Reticulocyte stains of peripheral blood from recipients of BM transduced with JAK2 WT (left) or JAK2 V617F (right). (E–F): Erythrocyte survival curves (E) and red cell mass determination (F) for the three groups (Balb/c background). The red cell mass of JAK2 V617F recipients was significantly greater than that of recipients of vector- or JAK2 WT-transduced BM (*P* = 0.0068 and *P* = 0.0009, respectively, unpaired *t*-test). (G) Plasma Epo levels for the three groups (B6 background). The mean Epo level (bar) of JAK2 V617F recipients was significantly lower than that of recipients of vector- or JAK2 WT-transduced BM (*P* = 0.0048 and *P* = 0.0151, respectively, unpaired *t*-test). (H) Number of CFU-E colonies from normal BM (green) or spleen of JAK2 V617F recipients (red), expressed as percent of maximal colony number (Balb/c background). Data for JAK2 WT recipients were similar to normal mice (data not shown). All studies were carried out between 6 and 10 weeks post-transplantation.

To investigate the molecular pathogenesis of MPD induced by JAK2 V617F, we expressed murine JAK2 WT or JAK2 V617F in BM of two different strains of mice, Balb/c and C57Bl/6 (B6), using retroviral transduction and transplantation [21]. In both strains, recipients of JAK2 V617F-transduced BM exhibited markedly increased blood hematocrit and hemoglobin levels that were evident by three weeks after transplantation and sustained for months (Figure 1A and 1B), while recipients of JAK2 WT- or vector-transduced BM were normal. Polycythemia was accompanied by a striking increase in circulating reticulocytes (Figure 1C and 1D). The erythrocyte lifespan in JAK2 V617F recipients was normal (Figure 1E), and polycythemia was accompanied by a proportional increase in red cell mass (Figure 1F). These results indicate that the polycythemia in these recipients was due to primary overproduction of erythrocytes, not to an increase in red cell survival or decrease in plasma volume. Plasma Epo levels were suppressed in JAK2 V617F recipients (Figure 1G), demonstrating that the erythropoiesis in these mice is autonomous, and not driven by overproduction of Epo. CFU-E from BM or spleen of these recipients showed increased sensitivity to Epo, with about 25–30% of CFU-E growing in the absence of exogenous Epo (Figure 1H), demonstrating the presence of EEC in the polycythemic mice. Interestingly, the frequency of Epo-independent BFU-E in these recipients was much lower (data not shown), suggesting that JAK2 V617F acts in the most differentiated erythroid progenitors to promote Epo-independent maturation. Together, these results indicate that JAK2 V617F expression directly induces polycythemia in mice through Epo-independent overproduction of erythrocytes. The fact that the central erythroid features of PV are recapitulated by expression of JAK2 V617F argues that it is the primary and direct cause of human PV.

### JAK2 V617F induces strain-dependent leukocytosis, but not thrombocytosis

**Figure 2:**
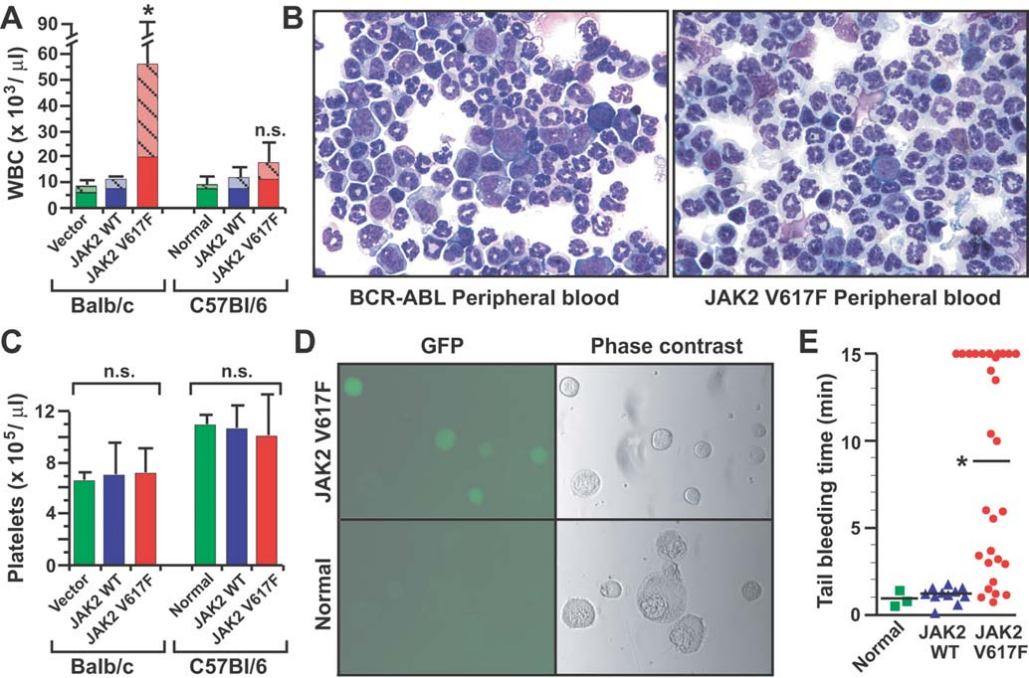
Effect of JAK2 V617F on leukocyte and platelet counts. (A) Peripheral blood leukocyte counts for the three cohorts in Balb/c and B6 backgrounds as in Figure 1. The difference in leukocyte counts between JAK2 V617F and JAK2 WT recipients was significant for the Balb/c cohort (*P* = 0.0039) but not for B6 (*P* = 0.0542, unpaired *t*-test). The hatched portion of the histogram represents the percentage of the total leukocyte count comprised by neutrophils. (B) Wright-Giemsa-stained cytospins of peripheral blood leukocytes from representative mice with BCR-ABL-induced CML-like MPD (left) or JAK2 V617F-induced PV-like MPD (right). Note the predominance of mature neutrophils and lack of immature myeloid elements (myelocytes and promyelocytes) in the JAK2 V617F recipient. (C) Platelet counts for the groups in (A). There was no significant difference in platelet counts between the three cohorts in either stain. (D) GFP fluorescence (left panels) and phase contrast images (right panels) of purified megakaryocytes from recipients of JAK2 V617F-transduced BM (top panels) or normal control mice (bottom panels). Note the GFP fluorescence in the majority of megakaryocytes from JAK2 V617F recipients. (E) Tail bleeding time (Balb/c cohort) for the three groups. The mean bleeding time (bar) of JAK2 V617F recipients was significantly longer than that of vector or JAK2 WT recipients (*P* = 0.0328 and *P* = 0.0002, respectively, unpaired *t*-test).

In contrast to the polycythemia, the effects of JAK2 V617F expression on leukocyte and platelet counts were more variable. JAK2 V617F induced significant leukocytosis in Balb/c mice, but only a modest increase in B6 mice (Figure 2A). The majority of the leukocytes were mature neutrophils with some immature erythroid cells (Figure 2A and 2B), and there was a notable absence of circulating immature myeloid progenitors that are characteristic of BCR-ABL-induced CML-like MPD (Figure 2B). These results suggest that genetic differences between the two mouse strains affect the leukocytosis induced by JAK2 V617F. JAK2 V617F expression did not affect the platelet count in either strain (Figure 2C), despite evidence of proviral expression of GFP in megakaryocytes (Figure 2D). However, JAK2 V617F recipients had a marked defect in platelet function, with significantly prolonged tail bleeding time (Figure 2E). The lack of thrombocytosis suggests that additional events may be required for JAK2 V617F to cause ET, but qualitative platelet abnormalities induced by JAK2 V617F may contribute to the hemostatic complications of PV.

### Pathological characterization of MPD induced by JAK2 V617F

**Figure 3:**
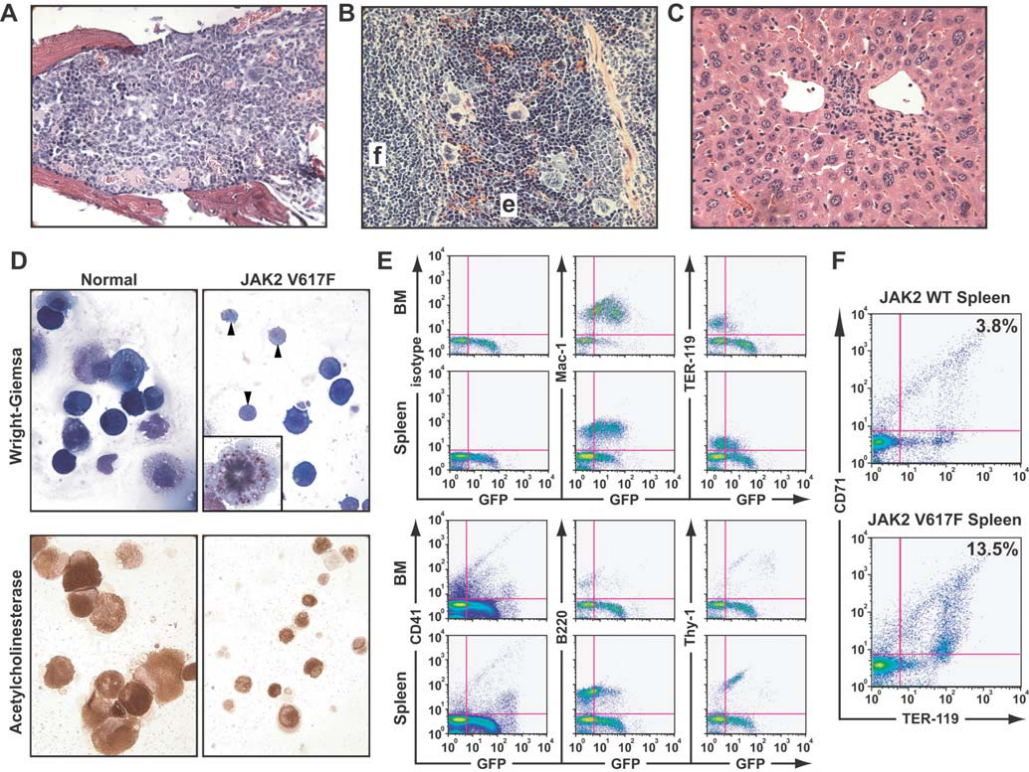
Histopathological characterization of the MPD induced by JAK2 V617F. (A–C): Hematoxylin and eosin stains (magnification 500×) of BM (A), spleen (B), and liver (C) from representative polycythemic recipients of JAK2 V617F-transduced BM. In the spleen, a lymphoid follicle (f) and an area of erythroid hyperplasia (e) are indicated. The same tissues from recipients of JAK2 WT-transduced BM showed no pathological changes compared with normal controls (data not shown). (D) Wright-Giemsa stains (top panels) and acetylcholinesterase stains (bottom panels) of purified megakaryocytes from recipients of JAK2 V617F-transduced BM (right panels) or normal control mice (left panels). Note the preponderance of small megakaryocytes from JAK2 V617F recipients, some of which are undergoing proplatelet formation (arrowheads), accompanied by abnormal mitoses with low apparent ploidy (insert). (E) Flow cytometric analysis of BM and spleen from a representative recipient of JAK2 V617F-transduced BM, stained with the indicated hematopoietic lineage antigens. The mean fluorescence intensity of GFP expressed from JAK2 retroviral provirus was reproducibly and significantly lower than the GFP fluorescence of a comparable BCR-ABL retrovirus (data not shown). Note the shift of GFP^+/lo^ erythropoiesis (TER-119^+^) from BM to spleen and the expression of GFP in low abundance CD41^+^ megakaryocytes. (F) Flow cytometric assessment of erythrocyte differentiation [47], assessed by expression of transferrin receptor (CD71) and TER-119 in splenocytes of recipients of JAK2 WT-transduced (top) and JAK2 V617F-transduced (bottom) BM. Note the increased (3.5-fold) population of CD71^+/lo^TER-119^+^ erythroblasts in JAK2 V617F recipients.

Despite the marked polycythemia, the MPD induced by JAK2 V617F was non-fatal in both strains, with recipients surviving for many months. By contrast, BCR-ABL-induced CML-like MPD is rapidly fatal in mice due to massive infiltration of lungs, liver, and spleen with maturing neutrophils [21]. At autopsy, JAK2 V617F recipients had significant splenomegaly, particularly in Balb/c mice (mean spleen weight 0.58±0.19 g versus 0.08±0.01 for Balb/c recipients of JAK2 WT-transduced BM, *P* = 0.0023 by unpaired *t*-test), but no involvement of lymph nodes or thymus, and no pulmonary hemorrhages. The BM of JAK2 V617F recipients was hypercellular, with a predominance of myeloid over erythroid cells, and less than 5% blasts (Figure 3A). Spleens exhibited massively increased erythroid precursors with partial disruption of follicular architecture and infiltration with mature myeloid cells (Figure 3B). Livers showed moderate periportal extramedullary myeloerythropoiesis (Figure 3C), while lungs had only minimal myeloid infiltration (data not shown). There were increased numbers of abnormal megakaryocytes present in BM and spleen (Figure 3A and 3B). When isolated after in vitro culture in thrombopoietin, megakaryocytes from JAK2 V617F recipients were noticeably smaller than their counterparts from normal mice or JAK2 WT recipients (Figure 3D), with many micromegakaryotes undergoing proplatelet formation and mitosis. Flow cytometric analysis of BM and spleen (Figure 3E) demonstrated GFP expression in myeloid, erythroid, megakaryocytic, and lymphoid cells, with a shift in erythropoiesis to the spleen, and a relative increase in immature (CD71^+/lo^TER119^+^) erythroid progenitors (Figure 3F).

### Constitutive activation of JAK2 V617F and downstream signaling pathways in polycythemic mice

**Figure 4:**
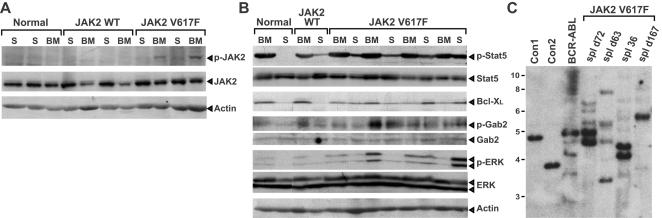
Constitutive activation of signaling pathways by JAK2 V617F. (A–B): Western blot analysis of primary myeloerythroid cell extracts from control (normal) mice and recipients of JAK2 WT- and JAK2 V617F-transduced BM. (A) Activated JAK2 (anti-pY1007/10008, top panel) and total JAK2 (middle panel) blots. Note that JAK2 V617F is modestly overexpressed and constitutively active. Anti-actin immunoblot demonstrating equivalent protein loading is at the bottom. (B) Constitutive activation of Stat5, Gab2, and ERK, with overexpression of Bcl-X_L_ in spleen cells from JAK2 V617F recipients. Primary myeloerythroid cell extracts from mice in (A) were immunoblotted with the indicated antibodies against total or phosphorylated Stat5, Gab2, ERK, and against Bcl-X. (C) Oligo- to polyclonal MPD in recipients of JAK2 V617F-transduced BM. Genomic DNA from spleens of individual JAK2 V617F recipients sacrificed at the indicated times post-transplant were analyzed with a radioactive *GFP* probe that detects a distinct band from each proviral integration event. For comparison, DNAs from control Ba/F3 cell lines each containing a single provirus (Con1 and Con2) and spleen DNA from a representative mouse with BCR-ABL-induced CML-like MPD are included at left, along with DNA size markers in kb.

To determine the biochemical properties of JAK2 V617F in vivo, we performed western blot analysis of extracts from primary myeloerythroid cells from recipients of JAK2 V617F- and JAK2 WT-transduced BM. There was modest (2- to 3-fold) overexpression of JAK2 in both cohorts, relative to the level of endogenous JAK2 protein (Figure 4A). There was constitutive activation of JAK2 V617F in BM and spleen cells, as evidenced by phosphorylation at the activation loop tyrosines 1007 and 1008, whereas JAK2 WT was not detectably phosphorylated. We also analyzed several signaling pathways that are activated by Epo receptor (Figure 4B). There was increased phosphorylation of Gab2 and ERK in spleens and BM from JAK2 V617F recipients that were somewhat variable from sample to sample, but no consistent activation of Akt (data not shown). There was constitutive activation of Stat5 and detectable expression of the Stat5 target Bcl-X_L_ in BM of both normal and polycythemic mice, with increased Stat5 phosphorylation and Bcl-X_L_ in spleens from JAKV617F recipients, although this could in part reflect the increase in splenic myeloerythroid cells in these mice. These results suggest that JAK2 V617F activates several signaling pathways in primary murine hematopoietic cells. Southern blot analysis of proviral integration demonstrated that multiple proviral clones contributed to the MPD induced by JAK2 V617F (Figure 4C), similar to that observed for BCR-ABL-induced CML-like MPD [21].

### Progression of polycythemia to myelofibrosis

**Figure 5:**
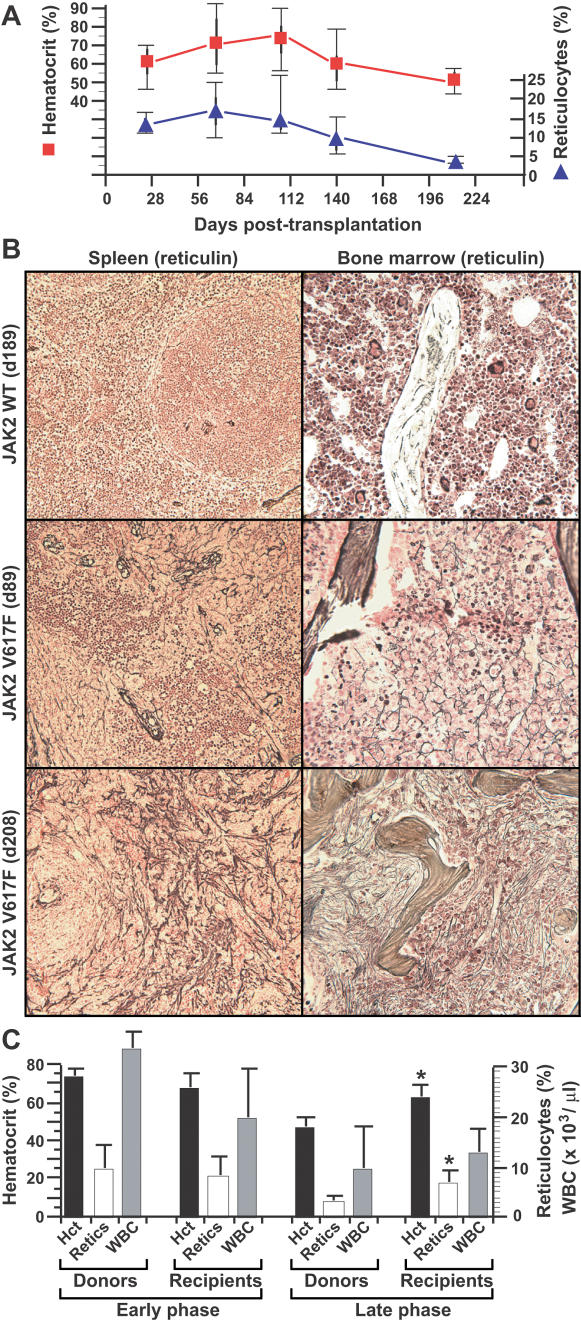
Evolution of JAK2 V617F-induced polycythemia to “spent” phase with myelofibrosis. (A) Box-style plots of hematocrit (red squares, left axis) and reticulocyte counts (blue triangles, right axis) in a cohort (*n* = 12) of Balb/c recipients of syngeneic JAK2 V617F-transduced BM, followed for over eight months after transplantation. Similar data were observed for a B6 cohort (data not shown). (B) Increasing fibrosis (demonstrated by reticulin staining) in spleen (left panels) and BM (right panels) of representative JAK2 V617F recipients at about 3 months (middle panels) and 7 months (bottom panels) after transplantation. Note the marked increase in reticulin staining at 7 months in the JAK2 V617F recipients, but not in recipients of JAK2 WT-transduced BM (top panels). (C): Efficient transfer of the PV-like MPD by transplantation of BM from primary mice sacrificed either in the early, polycythemic phase (left, *n* = 3, sacrificed 72–167 days post-transplant) or the late, myelofibrotic phase (right, *n* = 2, sacrificed 208 days post-transplant), to lethally irradiated syngeneic secondary recipients (*n* = 6 for early phase and *n* = 4 for late phase). The graphs depict mean hematocrit (black, left axis), reticulocyte count (white, right axis) and peripheral blood leukocyte count (grey, right axis) of the donors at the time of sacrifice, and of the recipients at day 30–70 post-transplant. For transplants performed in the late phase of the disease, the hematocrit and reticulocyte counts of recipients were significantly higher than of the donors (*P* = 0.0407 and *P* = 0.0337, respectively, unpaired *t*-test), while there was no significant difference between donors and recipients transplanted in the early phase.

The polycythemia induced by JAK2 V617F was maximal at 3 to 4 months post-transplantation, but tended to decrease with time in both strains, with hematocrit and reticulocyte counts returning to nearly normal ranges by 7 to 8 months after transplantation (Figure 5A), and some mice developing overt anemia. This coincided with a gradual but marked increase in fibrosis in the BM and spleen of JAK2 V617F recipients that was not observed in JAK2 WT recipients (Figure 5B). This is reminiscent of evolution of human PV to a “spent phase” resembling CIMF. Interestingly, polycythemia and reticulocytosis were efficiently resurrected in secondary mice by transplantation of BM and/or spleen cells from primary JAK2 V617F recipients, and this was equally true for donors harvested in the early, polycythemic phase of the disease as well as the later, myelofibrotic phase (Figure 5C). These results suggest that JAK2 V617F expression induces myelofibrosis, but the resulting impairment of erythropoiesis is due to a defect of the hematopoietic microenvironment, rather than a deficiency of malignant hematopoietic stem cells.

### JAK2 V617F-induced polycythemia is independent of Src kinases

**Figure 6:**
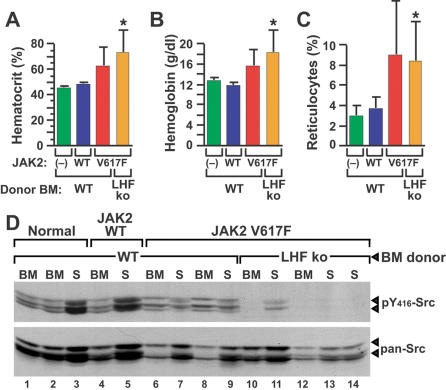
Polycythemia induced by JAK2 V617F is independent of Src kinases. (A–C): Hematocrit (A), blood hemoglobin (B), and reticulocyte counts (C) from normal (–) B6 mice (green), B6 recipients of B6 WT BM transduced with retrovirus expressing murine JAK2 WT (blue) or JAK2 V617F (red), and B6 *Lyn* ^−/−^ *Hck* ^−/−^ *Fgr* ^−/−^ BM transduced with retrovirus expressing JAK2 V617F (orange). The difference between recipients of JAK2 V617F-transduced *Lyn* ^−/−^ *Hck* ^−/−^ *Fgr* ^−/−^ BM and recipients of JAK2 WT-transduced WT BM was significant (unpaired *t*-tests) for hematocrit (*P* = 0.0009), hemoglobin (*P* = 0.0007), and reticulocytes (*P* = 0.0068), while the corresponding differences between recipients of JAK2 V617F-transduced BM from WT and *Lyn* ^−/−^ *Hck* ^−/−^ *Fgr* ^−/−^ donors were not significant. (D) Western blot analysis of extracts of primary myeloerythroid cells from individual normal (lanes 1–3) B6 mice, recipients of WT BM transduced with JAK2 WT retrovirus (lanes 4–5), recipients of WT BM transduced with JAK2 V617F retrovirus (lanes 6–9), and recipients of *Lyn* ^−/−^ *Hck* ^−/−^ *Fgr* ^−/−^ BM transduced with JAK2 V617F retrovirus (lanes 10–14). The membrane was immunoblotted with antibody recognizing the phosphorylated activation loop tyrosine (Y146 homolog) of c-Src, Lyn, Hck, Fyn, Lck, and Yes (top panel), and subsequently blotted with antibody recognizing total c-Src, Fyn, Yes, and Fgr (bottom panel).

This model of JAK2 V617F-induced polycythemia can be used to investigate signaling pathways critical for disease pathogenesis. Epo stimulation activates Src family kinases including Lyn [28], while a Src kinase inhibitor impaired the Epo-independent differentiation of erythroid progenitors from PV patients [12]. To investigate the role of Src kinases in the polycythemia induced by JAK2 V617F, we employed donor mice lacking Lyn, Hck, and Fgr, the three principal Src kinases in myeloerythroid progenitor cells [22], [29], as BM donors for transduction with JAK2 V617F. These mice have normal baseline hematopoiesis, but defective erythropoietic responses to stress [30]. Interestingly, recipients of *Lyn* ^−/−^ *Hck* ^−/−^ *Fgr* ^−/−^ BM transduced with JAK2 V617F developed polycythemia and reticulocytosis that tended to be greater than recipients of JAK2 V617F-transduced WT donor BM (Figure 6A–6C), although this did not reach statistical significance. The results demonstrate that these particular Src kinases are not required for polycythemia induced by JAK2 V617F, and might even play a negative role in JAK2 V617F signaling. There is a possibility that one or more of the other six vertebrate Src family kinases might compensate for lack of these three, particularly Fyn, Yes, and c-Src, which are expressed in myeloid cells. However, there was no overexpression of Fyn, Yes, or c-Src in myeloerythroid cells from polycythemic recipients of JAK2 V617F-transduced *Lyn* ^−/−^ *Hck* ^−/−^ *Fgr* ^−/−^ BM, and little or no detectable activation of these Src kinases, as assessed by antibody recognizing the phosphorylated activation loop tyrosine in Src (Figure 6D). This suggests that compensation by other Src family members is unlikely to play a role in polycythemia induced by JAK2 V617F in these mutant cells.

### JAK2 V617F-induced polycythemia responds to kinase inhibitor therapy

**Figure 7:**
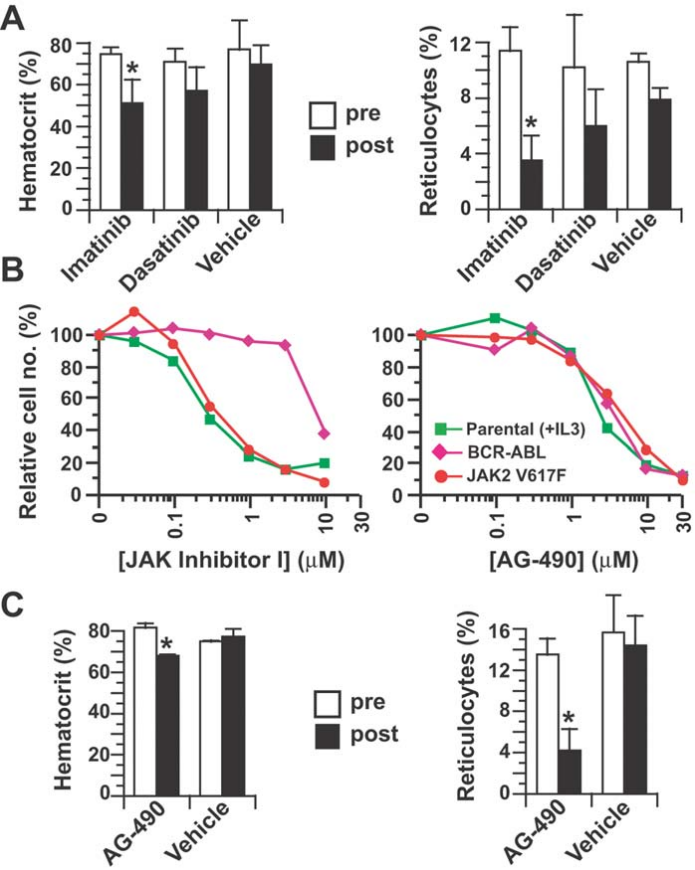
Polycythemia and reticulocytosis induced by JAK2 V617F responds to kinase inhibitor therapy. (A) Hematocrit (left panel) and reticulocyte counts (right panel) of cohorts of mice treated with twice daily oral gavage with 100 mg/kg imatinib (*n* = 3), 10 mg/kg dasatinib (*n* = 4), or vehicle (*n* = 2), determined before initiation of therapy (white bars, “pre”) or after 2 weeks of treatment (black bars, “post”). The hematocrit and reticulocyte count were significantly decreased in response to imatinib therapy (*P* = 0.0267 and *P* = 0.0053, respectively, unpaired *t*-test), while imatinib had no effect on these parameters in normal mice (data not shown). (B) Inhibition of Ba/F3 parental cells grown in IL-3 (green squares) or Ba/F3 cells expressing BCR-ABL (magenta triangles) or JAK2 V617F (red circles) grown without IL-3 by JAK Inhibitor I (left) or AG-490 (right). Note that both JAK Inhibitor I and AG-490 inhibit IL-3-dependent proliferation of parental Ba/F3 cells, but only AG-490 inhibits the growth of BCR-ABL-expressing cells, as previously reported [48]. (C) Hematocrit (left panel) and reticulocyte counts (right panel) of mice treated with continuous parenteral administration of 300 µg/day AG-490 (*n* = 3) or vehicle (*n* = 2), determined before initiation of therapy (white bars), or after 2 weeks of treatment (black bars). The hematocrit and reticulocyte count were significantly decreased in response to AG-490 therapy (*P* = 0.0134 and *P* = 0.0374, respectively, unpaired *t*-test). There was no significant effect of AG-490 on the hematocrit or reticulocyte count of recipients of JAK2 WT-transduced BM (data not shown).

We treated cohorts of polycythemic JAK2 V617F recipient mice for a 2-week period with small molecule tyrosine kinase inhibitors, including imatinib and the dual ABL/Src inhibitor dasatinib (BMS-354825). Imatinib therapy can reduce the hematocrit in some human PV patients, but has minimal effects on the level of JAK2 V617F [31]. Relative to vehicle-treated controls, imatinib-treated mice demonstrated significant decreases in hematocrit and reticulocyte counts, while the corresponding responses to dasatinib were less robust and did not reach statistical significance (Figure 7A). Imatinib therapy had no effect on hematocrit in normal mice, and neither drug decreased the percentage of circulating GFP^+^ leukocytes (data not shown). These results suggest that imatinib impairs JAK2 V617F-induced erythropoiesis through inhibition of a target other than ABL or c-Kit, and confirm that Src kinases may not be good targets for therapy in PV. There are no JAK2 inhibitors in clinical use, but the isoquinolinone compound JAK inhibitor I [15], [32] and the tyrphostin AG-490 [33] both inhibited the proliferation of Ba/F3 cells expressing JAK2 V617F with an IC_50_ of 0.3 and 3.5 µM, respectively (Figure 7B). AG-490 is not orally bioavailable, and parenteral administration is complicated by its short half-life and low solubility [33]. Nonetheless, continuous parenteral administration of AG-490 to polycythemic mice over a 2-week period caused a modest but significant decrease in hematocrit with a more pronounced drop in reticulocytes (Figure 7C), suggesting that chronic treatment with a JAK2 inhibitor would have therapeutic benefit in PV.

## Discussion

The JAK2 V617F mutation has been recently identified in most patients with PV, ET, and CIMF [13]–[16], [34]. While the initial frequency of the JAK2 V617F mutation reported in PV patients varied from 65–90%, upon the application of more sensitive sequencing methods, the mutation is detected in nearly every patient meeting rigorous clinical criteria for PV [35]. The mutant kinase has constitutive activity in cell lines [13], [15], and can transform IL-3 dependent hematopoietic cells such as Ba/F3 to cytokine independence [13], [15], [16]. This transformation activity may depend upon co-expression of a single-chain type I cytokine receptor, such as Epo receptor, G-CSF receptor, or thrombopoietin receptor [36]. In the initial report, retroviral expression of JAK2 V617F in murine BM induced erythrocytosis in transplanted recipient mice [13], but the syndrome was not characterized further.

Here, we have demonstrated that JAK2 V617F induces the complete spectrum of the clinicopathological features of human PV in mice, including polycythemia due to overproduction of erythrocytes, increased red cell mass, low plasma Epo levels, and presence of EEC in BM and spleen. Others have recently reported similar findings [37], [38]. Studies of familial PV have shown that the JAK2 V617F mutation is acquired somatically [39], [40], but have suggested that mutation in another gene may precede the JAK2 mutation in PV. However, the facts that polycythemia induced in mice by JAK2 V617F is polyclonal and manifested immediately following engraftment argue that JAK2 V617F is both necessary and sufficient for this phenotype. Increased expression of JAK2 V617F in patients with homozygous mutations may increase severity of the phenotype in PV [41], perhaps because the mutant and WT JAK2 kinases compete for Epo receptor binding [13]. In our model, robust polycythemia is induced by modest overexpression of JAK2 V617F in BM with two normal JAK2 genes, but further studies will be necessary to determine whether the phenotype is influenced by levels of normal JAK2. Taken together, these results establish JAK2 V617F as the direct cause of PV.

In contrast to the polycythemia, the effect of JAK2 V617F on the leukocyte and platelet counts was more variable. JAK2 V617F induced striking leukocytosis and neutrophilia in Balb/c mice, but leukocytosis was much milder in B6 recipients. This finding suggests that genetic differences between the two inbred strains modify the leukocyte response to the mutant JAK2 kinase. Somewhat surprisingly, there was no effect of JAK2 V617F on the platelet count in either strain, despite the detection of proviral gene expression (GFP) in CD41^+^ cells from spleen and BM, and in megakaryocytes purified from short-term in vitro cultures. This suggests that JAK2 V617F does not directly induce an increase in platelets, and that additional mutations or factors are necessary to explain the thrombocytosis in ET patients with JAK2 V617F. This is consistent with clinical studies demonstrating that the allelic ratio of the JAK2 V617F mutation does not account for the clonal hematopoiesis in many ET patients [42], and that ET patients with the JAK2 V617F mutation tend to have PV-like features [43]. Despite the lack of effect on platelet number, JAK2 V617F expression caused profound abnormalities of megakaryocyte maturation and platelet function, manifested by small megakaryocytes, abnormal mitoses during proplatelet formation, and a markedly prolonged tail bleeding time. These findings imply that JAK2 V617F might be the direct cause of the hemostatic abnormalities and mucosal bleeding frequently encountered in PV patients. We did not observe any obvious thrombotic events in our cohorts of polycythemic mice despite the significant elevation in hematocrit, and further studies will be necessary to determine if recipients of JAK2 V617F-transduced BM have abnormalities of coagulation.

The polycythemia induced by JAK2 V617F was sustained for several months, but eventually hematocrit and reticulocyte counts returned to normal levels in both strains by 8 months after transplantation. This was accompanied by prominent fibrosis in both BM and spleen of these recipients. This process bears a striking resemblance to the “spent” phase of PV, a condition resembling CIMF. CIMF patients with the JAK2 V617F mutation are clinically distinct from those lacking JAK2 mutation, and have a worse overall prognosis [44]. Our findings suggest that some or all CIMF patients with JAK2 V617F might actually represent PV patients that progressed to myelofibrosis prior to diagnosis. It is not clear whether the defective hematopoiesis in spent phase PV and CIMF is due to a deficiency of hematopoietic stem cells, or to a defective hematopoietic microenvironment. The fact that polycythemia can be efficiently transplanted to secondary recipients from mice with myelofibrosis argues that the problem is with the soil, not the seed [45].

This model system of human PV should be valuable for investigating the signaling pathways required for disease pathogenesis, and for testing the response to novel therapies, particularly drugs directed against specific molecular targets. In this report, we investigated the role of Src family kinases in the pathogenesis of JAK2 V617F-induced polycythemia. Although Src kinases are activated by JAK2 in erythroid cells upon Epo stimulation [28] and a Src inhibitor impaired Epo-independent maturation of PV erythroid progenitors [13], we demonstrated that polycythemia induced by JAK2 V617F was independent of Lyn, Hck, and Fgr, the three principal Src family kinases expressed in myeloerythroid cells. In complementary studies where diseased mice were treated with dasatinib, a potent inhibitor of both ABL and Src kinases, there was minimal response of polycythemia and reticulocytosis to a regimen that is very effective for treatment of mice inoculated with BCR-ABL-expressing cells [27]. These studies suggest that other Src family kinases are not compensating for deficiency of Lyn, Hck, and Fgr, and argue that therapy directed at Src kinases will be ineffective in PV patients. By contrast, imatinib therapy did cause a significant decrease in hematocrit and reticulocyte counts in JAK2 V617F recipients, and similar responses have been reported in PV patients [31]. The fact that imatinib was more effective than dasatinib indicates that a target other than ABL or c-Kit [46] mediates these responses. By analogy to CML, if PV is caused directly by JAK2 V617F, then the disease should be exquisitely sensitive to small molecule inhibitors of JAK2. There are no inhibitors of JAK kinases in current clinical use, and existing preclinical JAK2 inhibitors are compromised by lack of oral bioavailability and short in vivo half-life. Nevertheless, continuous parenteral delivery of the tyrphostin JAK2 inhibitor AG-490 to polycythemic mice caused a modest but significant drop in hematocrit, with a more profound decrease in reticulocyte count. These results provide proof-of-principle that more chronic administration of a potent, orally delivered JAK2 inhibitor should have therapeutic effects on the polycythemia, and perhaps the myelofibrosis and hemostatic abnormalities, suffered by MPD patients carrying the JAK2 V617F mutation. Further studies using this model should clarify the molecular pathogenesis of JAK2 V617F-associated MPD, and allow preclinical testing of novel therapies for these diseases.

## References

[1] Dameshek W (1951). Some speculations on the myeloproliferative disorders.. Blood.

[2] Van Etten RA, Shannon KM (2004). Focus on myeloproliferative diseases and myelodysplastic syndromes.. Cancer Cell.

[3] Daley GQ, Van Etten RA, Baltimore D (1990). Induction of chronic myelogenous leukemia in mice by the P210*^bcr/abl^* gene of the Philadelphia chromosome.. Science.

[4] Druker BJ, Talpaz M, Resta DJ, Peng B, Buchdunger E (2001). Efficacy and safety of a specific inhibitor of the BCR-ABL tyrosine kinase in chronic myeloid leukemia.. N Engl J Med.

[5] Spivak JL (2002). Polycythemia vera: myths, mechanisms, and management.. Blood.

[6] Prchal JF, Axelrad AA (1974). Letter: Bone-marrow responses in polycythemia vera.. N Engl J Med.

[7] Correa PN, Eskinazi D, Axelrad AA (1994). Circulating erythroid progenitors in polycythemia vera are hypersensitive to insulin-like growth factor-1 in vitro: studies in an improved serum-free medium.. Blood.

[8] Temerinac S, Klippel S, Strunck E, Roder S, Lubbert M (2000). Cloning of PRV-1, a novel member of the uPAR receptor superfamily, which is overexpressed in polycythemia rubra vera.. Blood.

[9] Moliterno AR, Hankins WD, Spivak JL (1998). Impaired expression of the thrombopoietin receptor by platelets from patients with polycythemia vera.. N Engl J Med.

[10] Xu MJ, Sui X, Zhao R, Dai C, Krantz SB (2003). PTP-MEG2 is activated in polycythemia vera erythroid progenitor cells and is required for growth and expansion of erythroid cells.. Blood.

[11] Parganas E, Wang D, Stravopodis D, Topham DJ, Marine JC (1998). Jak2 is essential for signaling through a variety of cytokine receptors.. Cell.

[12] Ugo V, Marzac C, Teyssandier I, Larbret F, Lecluse Y (2004). Multiple signaling pathways are involved in erythropoietin-independent differentiation of erythroid progenitors in polycythemia vera.. Exp Hematol.

[13] James C, Ugo V, Le Couedic JP, Staerk J, Delhommeau F (2005). A unique clonal JAK2 mutation leading to constitutive signalling causes polycythaemia vera.. Nature.

[14] Baxter EJ, Scott LM, Campbell PJ, East C, Fourouclas N (2005). Acquired mutation of the tyrosine kinase JAK2 in human myeloproliferative disorders.. Lancet.

[15] Levine RL, Wadleigh M, Cools J, Ebert BL, Wernig G (2005). Activating mutation in the tyrosine kinase JAK2 in polycythemia vera, essential thrombocythemia, and myeloid metaplasia with myelofibrosis.. Cancer Cell.

[16] Kralovics R, Passamonti F, Buser AS, Teo SS, Tiedt R (2005). A gain-of-function mutation of JAK2 in myeloproliferative disorders.. N Engl J Med.

[17] Jones AV, Kreil S, Zoi K, Waghorn K, Curtis C (2005). Widespread occurrence of the JAK2 V617F mutation in chronic myeloproliferative disorders.. Blood.

[18] Steensma DP, Dewald GW, Lasho TL, Powell HL, McClure RF (2005). The JAK2 V617F activating tyrosine kinase mutation is an infrequent event in both “atypical” myeloproliferative disorders and myelodysplastic syndromes.. Blood.

[19] Scott LM, Campbell PJ, Baxter EJ, Todd T, Stephens P (2005). The V617F JAK2 mutation is uncommon in cancers and in myeloid malignancies other than the classic myeloproliferative disorders.. Blood.

[20] Pear WS, Miller JP, Xu L, Pui JC, Soffer B (1998). Efficient and rapid induction of a chronic myelogenous leukemia-like myeloproliferative disease in mice receiving P210 bcr/abl-transduced bone marrow.. Blood.

[21] Roumiantsev S, Krause DS, Neumann CA, Dimitri CA, Asiedu F (2004). Distinct stem cell myeloproliferative/T lymphoma syndromes induced by ZNF198-FGFR1 and BCR-FGFR1 fusion genes from 8p11 translocations.. Cancer Cell.

[22] Meng F, Lowell CA (1997). Lipopolysaccharide (LPS)-induced macrophage activation and signal transduction in the absence of Src-family kinases Hck, Fgr, and Lyn.. J Exp Med.

[23] Friedman JS, Rebel VI, Derby R, Bell K, Huang T-T (2001). Absence of mitochondrial superoxide dismutase results in a murine hemolytic anemia responsive to therapy with a catalytic antioxidant.. J Exp Med.

[24] Ramakrishnan V, Reeves PS, DeGuzman F, Deshpande U, Ministri-Madrid K (1999). Increased thrombin responsiveness in platelets from mice lacking glycoprotein V.. Proc Natl Acad Sci USA.

[25] Drachman JG, Sabath DF, Fox NE, Kaushansky K (1997). Thrombopoietin signal transduction in purified murine megakaryocytes.. Blood.

[26] Wolff NC, Veach DR, Tong WP, Bornmann WG, Clarkson B (2005). PD166326, a novel tyrosine kinase inhibitor, has greater antileukemic activity than imatinib mesylate in a murine model of chronic myeloid leukemia.. Blood.

[27] Shah NP, Tran C, Lee FY, Chen P, Norrris D (2004). Overriding imatinib resistance with a novel ABL kinase inhibitor.. Science.

[28] Richmond TD, Chohan M, Barber DL (2005). Turning cells red: signal transduction mediated by erythropoietin.. Trends Biochem Sci.

[29] Hu Y, Liu Y, Pelletier S, Buchdunger E, Warmuth M (2004). Requirement of Src kinases Lyn, Hck and Fgr for *BCR-ABL1*-induced B-lymphoblastic leukemia but not chronic myeloid leukemia.. Nat Genet.

[30] Ingley E, McCarthy DJ, Pore JR, Sarna MK, Adenan AS (2005). Lyn deficiency reduces GATA-1, EKLF and STAT5, and induces extramedullary stress erythropoiesis.. Oncogene.

[31] Jones AV, Silver RT, Waghorn K, Curtis C, Kreil S (2005). Minimal molecular response in polycythemia vera patients treated with imatinib or interferon alpha.. Blood.

[32] Thompson JE, Cubbon RM, Cummings RT, Wicker LS, Frankshun R (2002). Photochemical preparation of a pyridone containing tetracycle: a Jak protein kinase inhibitor.. Bioorg Med Chem Lett.

[33] Meydan N, Grunberger T, Dadi H, Shahar M, Arpaia E (1996). Inhibition of acute lymphoblastic leukaemia by a Jak-2 inhibitor.. Nature.

[34] Zhao R, Xing S, Li Z, Fu X, Li Q (2005). Identification of an acquired JAK2 mutation in polycythemia vera.. J Biol Chem.

[35] Campbell PJ, Scott LM, Baxter EJ, Bench AJ, Green AR (2006). Methods for the detection of the JAK2 V617F mutation in human myeloproliferative disorders.. Methods Mol Med.

[36] Lu X, Levine R, Tong W, Wernig G, Pikman Y (2005). Expression of a homodimeric type I cytokine receptor is required for JAK2V617F-mediated transformation.. Proc Natl Acad Sci USA.

[37] Wernig G, Mercher T, Okabe R, Levine RL, Lee BH (2006). Expression of Jak2V617F causes a polycythemia vera-like disease with associated myelofibrosis in a murine bone marrow transplant model.. Blood.

[38] Lacout C, Pisani DF, Tulliez M, Gachelin FM, Vainchenker W (2006). JAK2V617F expression in murine hematopoietic cells leads to MPD mimicking human PV with secondary myelofibrosis.. Blood.

[39] Cario H, Goerttler PS, Steimle C, Levine RL, Pahl HL (2005). The JAK2V617F mutation is acquired secondary to the predisposing alteration in familial polycythaemia vera.. Br J Haematol.

[40] Bellanne-Chantelot C, Chaumarel I, Labopin M, Bellanger F, Barbu V (2006). Genetic and clinical implications of the Val617Phe JAK2 mutation in 72 families with myeloproliferative disorders.. Blood.

[41] Tefferi A, Lasho TL, Schwager SM, Strand JS, Elliott M (2006). The clinical phenotype of wild-type, heterozygous, and homozygous JAK2V617F in polycythemia vera.. Cancer.

[42] Levine RL, Belisle C, Wadleigh M, Zahrieh D, Lee S (2006). X-inactivation based clonality analysis and quantitative JAK2V617F assessment reveals a strong association between clonality and JAK2V617F in PV but not ET/MMM, and identifies a subset of JAK2V617F negative ET and MMM patients with clonal hematopoiesis.. Blood.

[43] Campbell PJ, Scott LM, Buck G, Wheatley K, East CL (2005). Definition of subtypes of essential thrombocythaemia and relation to polycythaemia vera based on JAK2 V617F mutation status: a prospective study.. Lancet.

[44] Campbell PJ, Griesshammer M, Dohner K, Dohner H, Kusec R (2006). V617F mutation in JAK2 is associated with poorer survival in idiopathic myelofibrosis.. Blood.

[45] Westervelt P, Ley TJ (1999). Seed versus soil: the importance of the target cell for transgenic models of human leukemias.. Blood.

[46] Schittenhelm MM, Shiraga S, Schroeder A, Corbin AS, Griffith D (2006). Dasatinib (BMS-354825), a dual SRC/ABL kinase inhibitor, inhibits the kinase activity of wild-type, juxtamembrane, and activation loop mutant KIT isoforms associated with human malignancies.. Cancer Res.

[47] Socolovsky M, Nam H, Fleming MD, Haase VH, Brugnara C (2001). Ineffective erythropoiesis in Stat5a(−/−)5b(−/−) mice due to decreased survival of early erythroblasts.. Blood.

[48] Xie S, Lin H, Sun T, Arlinghaus RB (2002). Jak2 is involved in c-Myc induction by Bcr-Abl.. Oncogene.

